# An Augmented Reality-Assisted Prognostics and Health Management System Based on Deep Learning for IoT-Enabled Manufacturing

**DOI:** 10.3390/s22176472

**Published:** 2022-08-28

**Authors:** Liping Wang, Dunbing Tang, Changchun Liu, Qingwei Nie, Zhen Wang, Linqi Zhang

**Affiliations:** College of Mechanical and Electrical Engineering, Nanjing University of Aeronautics and Astronautics, Nanjing 210016, China

**Keywords:** augmented reality, PSO-CNN, GRU-attention, RUL prediction, IoT-enabled manufacturing

## Abstract

With increasingly advanced Internet of Things (IoT) technology, the composition of workshop equipment has become more and more complex. Based on this, the rate of system performance degradation and the probability of fault have both increased. Owing to this, not only has the difficulty of constructing the remaining useful life (RUL) model increased but also the improvement in speed of maintenance personnel cannot keep up with the speed of equipment replacement. Therefore, an augmented reality (AR)-assisted prognostics and health management system based on deep learning for IoT-enabled manufacturing is proposed in this paper. Firstly, the feature extraction model based on Convolutional Neural Network-Particle Swarm Optimization (PSO-CNN) is proposed with the purpose of excavating the internal associations in large amounts of production data. Based on this, the high-accuracy RUL prediction is accomplished by Gate Recurrent Unit (GRU)-attention, which can capture the long-term and short-term dependencies of time series and successfully solve the gradient disappearance problem of RNN. Moreover, more attention will be paid to important content with the help of the attention mechanism. Additionally, high-efficiency maintenance guidance and visible instructions can be accomplished by AR. On top of this, the remote expert can offer help when maintenance personnel encounters tough problems. Finally, a real case was implemented in a typical IoT-enabled workshop, which validated the effectiveness of the proposed approach.

## 1. Introduction

In the wake of the continuous advancement of the Internet of Things (IoT) technology, the intelligence of the equipment in the manufacturing system has been continuously improved, and the structural composition has become more complex [1,2]. Meanwhile, due to the complicated and capricious operating conditions of the industrial equipment system and the relatively harsh operating environment, the rate of system performance degradation and the probability of failure have both increased [3,4]. On top of this, if an accident caused by failure occurs in key equipment systems, it can lead to significant economic losses and even casualties. With the purpose of preventing similar incidents, an investment of a huge amount of financial and human resources to improve the security and stability of the equipment system is needed. Relevant data shows that the cost of equipment maintenance in the field of manufacturing accounts for about 15–70% of its total production cost [5]. Due to this, it is of great importance to quickly and precisely predict the remaining useful life (RUL) of the system before serious accidents occur, especially in the early stage of degradation of equipment system performance. On the basis of this, it is of great significance to implement effective maintenance to guarantee the secure and dependable operation of the IoT-enabled manufacturing equipment.

Prognostics and Health Management (PHM) is an important technology for the comprehensive evaluation of modern mechanical equipment. A reasonable PHM system can accurately monitor the operating status of mechanical equipment and provide an effective maintenance scheme. In addition, it can also predict reliability and RUL to prevent the occurrence of the fault. Based on this, maintenance costs will be reduced, and production efficiency will be improved. Deep learning has a strong, nonlinear function fitting ability and can better capture the implicit functional relationship in time series data [6]. Therefore, deep learning-based algorithms have become a hot spot in PHM research in recent years.

In terms of the fault feature extraction, Deutsch et al. [7] used the dataset of six vibration state indicators (e.g., root mean square and frequency modulation characteristics) as the health indicators of fault characteristics, which are the input of the DBN network and applied to the RUL prediction of bevel gears. Jia et al. [8] used spectral information as the input features of Deep Neural Networks (DNNs). Liao et al. [9] extracted the original time and frequency domain features of 21 rolling bearings as the input features of the Enhanced Restricted Boltzmann Machine (ERBM) for the RUL prediction of the bearing. Li et al. [10] put forward a multimodal deep support vector classification method to excavate time, frequency, and time-frequency feature sets from vibration signals. Additionally, Gaussian–Bernoulli is applied in DBM to each feature set individually to diagnose gear faults. In terms of RUL prediction, Guo et al. [11] proposed a feature fusion method based on Long Short-term Memory Network (LSTM) to fuse multiple feature data and construct a health factor for bearing RUL prediction. Based on this, high prediction accuracy was obtained. Zhu et al. [12] developed a new deep feature learning method, which used time-frequency representation and a multi-scale convolutional neural network to predict the remaining life and achieved high prediction accuracy. Wang et al. [13] extracted the time-varying features of the vibration signal through correlation analysis and then used the three-layer feedforward neural network algorithm to model the time-varying features of the vibration signal. Finally, the predicted time-varying features of the vibration signal were input to the scale. The survival rate and hazard rate of the bearing are estimated in the risk model, and the RUL of the bearing is predicted. Experiment results proved the validity and reliability of the method for RUL prediction. Elsisi et al. [14] proposed a fault diagnosis method for power transformers, which was driven by a one-dimension convolutional neural network (CNN). On the basis of this, the proposed method could resist uncertainties and achieve robustness. A novel ensemble long short-term memory neural network (ELSTMNN) model for RUL prediction was proposed by Cheng et al. [15] to improve both the accuracy of RUL prediction and the generalization ability in different prediction scenarios. Kong et al. [16] conducted research on the basis of the combination of CNN and LSTM (CNN-SLTM) and achieved good results in engine RUL prediction. Liu et al. [17] proposed a combination of a bi-directional long short-term memory network (Bi-LSTM) and convolutional neural network (CNN) to predict the remaining useful life of aero-engines. Based on this, long-term temporal dependencies and important local features from sequence data can be captured. Ellefsen et al. [18] proposed an RBM-LSTM-enabled method to predict the RUL of the turbofan engine. On the basis of this, degraded features can be extracted by RBM, and degraded features can be processed by LSTM. Zhang et al. [19] proposed a novel RUL prediction method combined with CNN and XGBoost, and the RUL result can be predicted by CNN and XGBoost, respectively. Based on this, the final RUL result can be obtained by weighting the predicted results.

Although the existing research has achieved certain results, the following shortcomings need to be further improved.

(1) Although the above RUL prediction methods have achieved good theoretical results, most of them still rely on professional signal processing knowledge and the experience of the related domain expert in practical application scenarios, especially for machines with different degradation modes. Meanwhile, manual participation in feature engineering makes it difficult for the modeling method to have generality. Considering that the equipment operates under complex working conditions, the aforementioned modeling difficulty will be multiplied. Owing to this, it is essential to develop a model which can automatically complete the extraction of effective features from complex signals and have certain universality instead of manually annotated features.
(2) The current maintenance method is single, mainly relying on paper maintenance manuals and the existing experience of maintenance personnel. The learning speed of maintenance personnel cannot keep up with the intelligent upgrading speed of equipment in the manufacturing system, which will lead to low maintenance efficiency and frequent misoperation.

In order to address the problems mentioned above, an augmented reality-assisted PHM based on deep learning for IoT-enabled manufacturing is proposed in this paper. The main contributions can be summarized in the following two aspects.

(1) The feature extraction model based on PSO-CNN is developed to excavate the internal associations in the vast sea of production data. Meanwhile, the RUL prediction is accomplished by GRU-attention. Based on this, the long-term and short-term dependencies of time series can be captured, and the gradient disappearance problem can be successfully solved. Due to this, the high-accuracy prediction result can be achieved with the help of the combination between GRU and the attention mechanism.
(2) Augmented reality is applied for generating the immersive scene to achieve high-efficiency maintenance guidance, and the visible instructions can be integrated into the physical world. Additionally, maintenance personnel can get help from a remote expert when meeting tough problems that can not be solved alone.

The remainder of this paper is structured as follows. Section 2 systematically describes the related work in terms of prognostics and health management systems, deep learning in RUL, and augmented reality-assisted maintenance. In Section 3, the framework architecture of the proposed approach is constructed. Section 4 describes the implementation of the framework based on the deep learning-enabled RUL prediction model and augmented reality-assisted maintenance. In Section 5, a real case is implemented in a typical IoT-enabled manufacturing workshop, and the experiment’s results are analyzed. Section 6 further verifies the performance of the proposed approach by implementing comparative experiments. Finally, Section 7 presents the conclusion and outlook.

## 2. Literature Review

### 2.1. Deep Learning in PHM

RUL prediction is a pivotal step in the implementation of PHM technology, which can analyze the relevant information of the current operating state of the equipment through the measurement data monitored by the equipment at various times. Additionally, the relevant factors that affect the equipment degradation trend can be extracted. Based on this, the future state can be predicted to obtain the fault time of the equipment [20].

Along with the evolution of deep neural network theory, deep learning makes it possible to train deep models, which can strengthen the deep feature extraction ability and the complex nonlinear expression ability [21]. The structural difference of deep neural network models is the root cause that affects the precision of the RUL prediction, such as the network type, the training algorithms, and so on [22,23]. Tamilselvan et al. [24] firstly proposed a health state classification model driven by Deep Belief Network (DBN), which has been applied to aero-engine fault diagnosis. Li et al. [25] proposed a one-dimensional CNN model to process multi-dimensional sensor data for RUL prediction. Liu et al. [26] applied CNN-LSTM to predict the RUL of machine tools in the workshop, in which fault features were extracted through CNN, and the RUL prediction curve was fitted with LSTM. The experiment results indicated that the suggested approach in the literature has high precision of RUL prediction. Babu et al. [27] applied CNN to evaluate the RUL through the data acquired by various sensors and proposed a regression method based on a deep convolutional neural network to predict the RUL of mechanical equipment. Based on this, a multi-layer CNN model was constructed, and the convolution and pooling operations were performed in the time dimension. Finally, the method was compared with several algorithms on two public datasets, and the efficacy of the approach was proved. Yang et al. [28] proposed an RUL prediction approach for mechanical equipment based on a double CNN model framework, which could receive the original vibration signal by way of the first CNN model and determine the initial fault point of the original signal. Afterward, the second CNN model estimated the RUL of the original signal through the state value in the degradation model corresponding to the initial estimation point. Finally, comparative experiments were conducted on four bearing degradation datasets, which proved that the suggested approach has high precision of prediction and robustness. Zhang et al. [29] modeled the time characteristics of sensor data, and developed a RUL prediction algorithm driven by RNN. Moreover, a variety of models were combined to accomplish the prediction of the RUL.

However, when the RNN calculates the gradient and updates the network, the RNN algorithm will have the problem of gradient disappearance or gradient explosion, which means that the RNN network cannot obtain the long-term relationship in the data. Owing to this, the actual performance of RNN in RUL prediction applications will be greatly affected. To sum up, the PHM of equipment systems under complex working conditions is a scientific problem with great practical background and important theoretical significance. The key to solving this problem is to predict the RUL of the system on the basis of the condition monitoring data.

### 2.2. Technical Support Based on Augmented Reality

The application of the head-mounted display (HMD) frees the user’s hands and can guide the user during the maintenance process. HMD viewing information is natural and intuitive, with a strong sense of immersion, and it is convenient for users to perform human–computer interaction through gestures, voice, and virtual buttons [30]. At present, emerging augmented reality (AR) glasses on the market have integrated video synthesis penetrating and optical principle penetrating HMDs (e.g., Microsoft Hololens), which can provide users with all-around assistance in maintenance [31].

In the early stage of augmented reality-assisted maintenance, manual placement of signs was mainly used to achieve target tracking technology. However, when some marking methods were applied in maintenance workshops, the tracking registration was easily lost due to the influence of the workshop environment. With the iterative development of hardware systems, the computing power has been significantly improved, and the shortcomings of the marking method in maintenance have gradually become prominent. Researchers have begun to apply markerless technology to augmented reality-assisted maintenance systems. Wu et al. [32] proposed a brilliant fault prediction system driven by AR, which used the hierarchical correlation between big data and operating faults, feature extraction of operating faults, and intelligent diagnosis of operating faults to construct a dynamic prediction model of faults. This model established an active maintenance system, which improved production efficiency and ensured production safety. Aqueveque et al. [33] introduced a vibration sensor network based on AR, and the machine operating states can be can accurately classified with an accuracy rate of between 85% and 95%. Ooi et al. [34] proposed a parameter-free vibration analysis technique based on AR to track the operating state of industrial exhaust fans by clustering and classifying the types of vibrations generated by the machine. Gunda et al. [35] proposed an operation and maintenance method based on AR, which was applied to an inverter. Based on this, a large number of maintenance records of photovoltaic owners and operators can be collected, and the common failure modes of inverters can be easily understood with the help of AR. Leonardi et al. [36] developed a risk maintenance approach based on AR to design detection tasks assigned to service robots in wind power plants. The proposed approach can automatically generate preventive maintenance mechanisms that rely on real-time data to dynamically adjust the priority of interventions. Li et al. [37] took the design and implementation of a log analysis system for operation and maintenance as an example and expounded the use of AR technology to analyze and mine maintenance data to improve operation and maintenance plans. In terms of intelligent monitoring of operation and maintenance systems, Liang et al. [38] proposed optimization of the loss function and image synthesis in an AR-based intelligent computer room monitoring system.

Augmented reality, as an emerging technology that integrates virtual and reality, will store various information about workshop equipment in the real environment in the form of virtual information. Additionally, the status of various equipment will be clear at a glance. Based on this, the operation and maintenance personnel can actually carry out maintenance operations on the machine through virtual instructions.

### 2.3. Research Gaps

(1) Although the above several classic deep learning models have been implemented in the field of PHM, most of the existing algorithms still rely on manual feature extraction. However, RUL prediction results lack uncertainty expression, which will result in poor performance of the existing RUL models. In this regard, an RUL prediction method combining PSO-CNN and GRU-attention is developed to improve the efficiency of feature extraction and the accuracy of RUL prediction.
(2) The existing maintenance methods mostly rely on paper maintenance manuals and the existing experience of maintenance personnel, which will result in low maintenance efficiency and frequent misoperation. With the help of AR, visible maintenance instructions can be integrated into the physical maintenance environment, which can not only strengthen the competence of the maintenance personnel but also save training time for new personnel.

## 3. Framework Architecture

**Figure 1:**
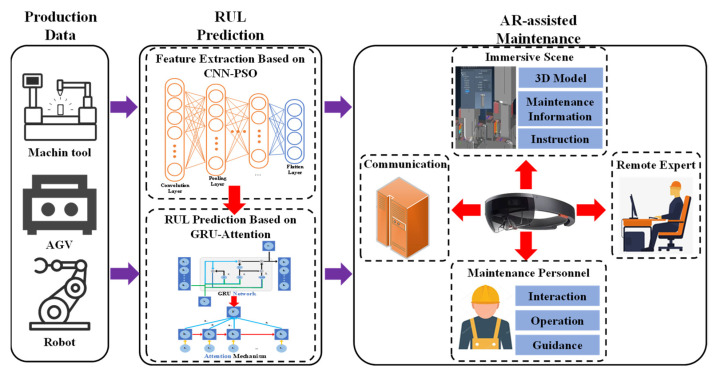
Framework architecture of the augmented reality-assisted PHM system driven by deep learning.

The framework architecture of the augmented reality-assisted PHM system driven by deep learning is described in Figure 1, which consists of the production data module, the RUL prediction module, and the AR -assisted maintenance module. The specific functions of these modules are described minutely as follows.

(1) ***Production data module:*** This module is responsible for production data acquisition from machine tools, AGVs, and robots. Afterward, the production data can be preprocessed through data cleaning and data normalization. The data cleaning is responsible for excluding abnormal data (e.g., the noise data during the acquisition of sensors). Meanwhile, the data normalization can transform a dimensional expression into a dimensionless expression and become a scalar. Based on this, the model can extract key information and avoid the interference of useless information.
(2) ***RUL prediction module:*** In order to excavate the internal associations in the vast sea of data, the feature extraction model based on PSO-CNN is proposed. CNN is responsible for extracting the important factors through convolution and pooling operations. Accordingly, the fault feature extraction model based on CNN is optimized by using the optimization feature of PSO to improve the feature extraction efficiency. Based on the high-efficiency feature extraction, the RUL prediction is accomplished by GRU-attention. The high-accuracy prediction result can be achieved with the help of the combination between GRU and the attention mechanism.
(3) ***AR-assisted maintenance:*** With the purpose of achieving high-efficiency maintenance guidance, AR is applied to generate the immersive scene, which consists of a 3D model, the maintenance information, and the instruction. Through the WebRTC communication mechanism, maintenance personnel can get help from a remote expert when meeting tough problems that can not be solved alone. With the help of visible guidance, maintenance personnel can interact with the immersive scene and accomplish the maintenance tasks well.

## 4. Implementation of the Framework

### 4.1. Construction of the Remaining Useful Life

For the target device, assuming that the degradation data $x_{1:k}=\left\{x_{1},x_{2},\ldots ,x_{k}\right\}$ is monitored at time $t_{k}$, and $X\left(t_{k}+l\right)$ is the performance degradation at future time $t_{k}+l$ (l≥0), which is estimated at the current time $t_{k}$. Then the remaining life $T_{RUL}$ of the target device can be defined as the time l when the performance parameter $X\left(t_{k}+l\right)$ presses close to the fault threshold $D_{f}$ for the first time at the current time $t_{k}$, which are formulated as follows:

(1) $$T_{RUL}=\inf\{l:X(t_{k}+l_{k}\}\geq D_{f}|X\left(t_{0}\right)<D_{f}\}$$

When the random parameters are $\mu_{\beta ,k}$ and $\sigma_{\beta ,k}$ at the current time $t_{k}$, the remaining life probability density function of the target equipment is described as follows:

(2) $$f_{T_{RUL}}(l_{l}|x_{1:k})≅\frac{1}{A^{*}}g_{T_{RUL}}(l_{l}|x_{1:k})$$

(3) $$A^{*}=\int_{0}^{+\infty }g_{T_{RUL}}\left(u\right)du$$

(4) $$\begin{array}{c} g_{T_{RUL}}\left(l_{l}|x_{1:k}\right)\\ =\frac{\gamma d_{2}}{\left(d_{1}+d_{2}t\right)\sqrt{2\pi \left\{{\left[d_{1}+d_{2}\left(t_{k}+l_{k}\right)\right]}^{\gamma }+\sigma_{\beta }^{2}{\left(t_{k}+l_{k}\right)}^{2}\right\}}}\\ \times \exp\left\{-\frac{{\left(D_{f}-x_{0}-\mu_{\beta ,k}\left(t_{k}+l_{k}\right)\right)}^{2}}{2\left[({\left(d_{1}+d_{2}\left(t_{k}+l_{k}\right)\right)}^{\gamma }+\sigma_{\beta ,k}^{2}{\left(t_{k}+l_{k}\right)}^{2}\right]}\right\}\\ \times \left\{\begin{array}{c} D_{f}-x_{0}-\left[t_{k}+l_{k}-h\left(t_{k}+l_{k}\right){\left(d_{1}+d_{2}\left(t_{k}+l_{k}\right)\right)}^{\gamma }\right]\\ \times \frac{\left(D_{f}-x_{0}\right)\sigma_{\beta ,k}^{2}\left(t_{k}+l_{k}\right)+\mu_{\beta ,k}{\left(d_{1}+d_{2}\left(t_{k}+l_{k}\right)\right)}^{\gamma }}{\left[{\left(d_{1}+d_{2}\left(t_{k}+l_{k}\right)\right)}^{\gamma }+\sigma_{\beta ,k}^{2}{\left(t_{k}+l_{k}\right)}^{2}\right]} \end{array}\right\} \end{array}$$

where μ, σ, $d_{1}$, $d_{2}$ and γ are the unknown parameters that need to be evaluated, respectively.

Therefore, the allocation function of the target machine can be described by the numerical integration as follows:

(5) $$F_{T}\left(t\right)=P\left\{T\leq t\right\}=\int_{o}^{t}f_{T}\left(u\right)du$$

### 4.2. RUL Prediction Based on Deep Learning

**Figure 2:**
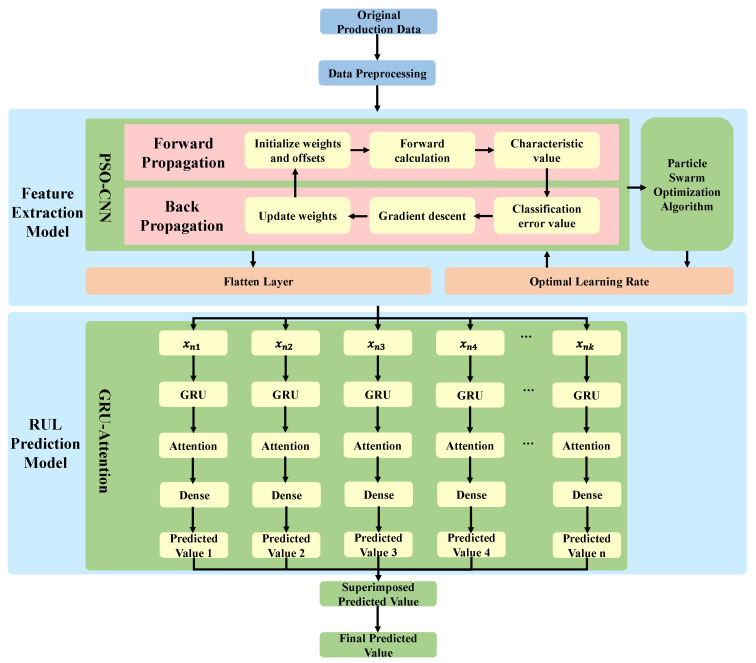
The procedure of RUL prediction driven by PSO-CNN and GRU-Attention.

For the purpose of improving the efficiency and accuracy of the RUL prediction model, a deep learning-enabled approach is proposed in this section. The procedure of RUL prediction based on PSO-CNN and GRU-Attention is described minutely in Figure 2, and the specific illustration is shown below.

(1) Data input: The production data is preprocessed and sampled first, and the one-dimensional production data is the input of CNN. Based on this, CNN can better identify the signal
(2) Feature extraction model: Firstly, feature extraction is performed on the production data through the convolutional layer in the CNN forward propagation. On top of this, the pooling layer can reduce the dimension of the feature data. Additionally, the fault feature extraction model based on CNN is optimized by using the optimization feature of PSO to improve the feature extraction efficiency.
(3) RUL prediction model: GRU is used to fit associations between features mined by CNN. Based on the attention mechanism, more user attention will be paid to vital content. Therefore, the accuracy of the RUL prediction model will be improved.

#### 4.2.1. Fault Feature Extraction Based on PSO-CNN

Firstly, forward propagation is performed by CNN, including convolution and pooling operations. On the basis of this, the calculation of the convolution layer is formulated as follows:

(6) $$x_{j}^{n}=f\left(\sum_{i\in M_{j}}x_{i}^{n-1}K_{ij}^{n}+b_{j}^{n}\right)$$

where $M_{j}$ is the input of production data, $x_{j}^{n}$ is the eigenvalue j of the n-th layer. $K_{ij}^{n}$ is the convolution kernel function, and f() is the activation function. Besides $b_{j}^{n}$ is the bias function.

The convolutional layer and the pooling layer are alternately calculated. The convolutional layer is followed by the pooling layer. The calculation for the pooling layer is described as follows:

(7) $$x_{j}^{n+1}=f\left(\sum_{j}x_{j}^{n}\omega_{j}^{n+1}+b_{j}^{n+1}\right)$$

where $\omega_{j}^{n}$ is the weight of the pooling layer.

The result obtained by the output layer can be expressed as below:

(8) $$y=f_{n}\left(\cdots \left(f_{2}\left(f_{1}\left(x\cdot \omega^{1}\right)\omega^{2}\right)\right)\cdots \right)\omega^{n}$$

The error function is expressed as follows:

(9) $$E=\frac{1}{n}\sum_{i=1}^{N}\sum_{j=1}^{C}{\left(y_{ji}^{d}-y_{ji}\right)}^{2}$$

PSO is used to find the optimal value by initializing a group of particles and continuously updating the speed and position. Firstly, the PSO needs to be initialized as follows:

(10) $$Y_{i}\left(0\right)=Y_{max}-\alpha \left(Y_{max}-Y_{min}\right)$$

(11) $$x_{i}\left(0\right)=0.1S+R$$

where $Y_{i}\left(0\right)$ is the initial velocity of particle i, $Y_{max}$ and $Y_{min}$ are the upper and lower limits of particle velocity, respectively. α is a random number in (0, 1), and $x_{i}\left(0\right)$ is the initial position of particle i. S is random number in (−1, 1), and R is the value selected according to human experience before the parameters are optimized.

The position and velocity update formulas are respectively expressed as below:

(12) $$x_{i}\left(t+1\right)=x_{i}\left(t\right)+Y_{i}\left(t+1\right)$$

(13) $$Y_{i}\left(t+1\right)=wY_{i}\left(t\right)+h_{1}R_{1}\left(P_{i}-x_{i}\left(t\right)\right)+h_{2}R_{2}\left(P_{i}-x_{i}\left(t\right)\right)$$

where $h_{1}$ and $h_{2}$ represent acceleration factors, which are constants. Meanwhile, w is inertia factor. $R_{1}$ and $R_{2}$ represent accidental numbers generated in (0, 1). Additionally, $x_{i}\left(t\right)$ is the position of particle *i* in *t*-th iteration, and $Y_{i}\left(t\right)$ is the velocity of particle *i* in *t*-th iteration.

The steps to optimize CNN parameters using PSO are described as follows (Algorithm 1).

(1) Initialize particle parameters.
(2) Set the value range of the parameters that CNN needs to optimize and use it as the range interval for updating the particle speed and position. The particles exceeding the value range take the maximum or minimum value of the interval.
(3) Train the CNN model and output the accuracy.
(4) If the training meets the accuracy requirements or the number of iterations, use the obtained parameters as the optimal structural parameters of the CNN; otherwise, return to step (3).
(5) Use the optimal parameters to test the CNN to obtain the feature extraction results of equipment fault signals in the IoT manufacturing workshop.

**Algorithm 1:** Hyperparametric optimization algorithm of PSO-CNN.Input: MAX_Iteration, boundary of the hyperparametric space *θ*, number of particles *n*Output: Optimal super parameters and maximum fault feature recognition accuracy1 Randomly generate *n* particles to form the initial population:

$\theta_{j}=\left\{cnn_{L},ci,k,d,r\right\},\;\;j=1,2,\ldots ,n$

Set an optimal super parameter ($\theta_{best}$) and maximum accuracy (*accuracy_best*)2 for *I* = 1:MAX_Iteration3   for *j* = 1: *n*4    train CNN model and output accuracy5    *accuracy* ← execute (CNN model)6    if *accuracy* > *accuracy_max*7      $\theta_{best}\leftarrow \theta_{j}$
8      *accuracy_best* ← *accuracy*9 Update the speed and position of each particle

#### 4.2.2. RUL Prediction Based on GRU-Attention

The GRU network model is an improved recurrent neural network (RNN), which has the ability to capture the long-term and short-term dependencies of time series and successfully solve the gradient disappearance problem of RNN. The internal structure is simple, and the training parameters are reduced while ensuring the prediction accuracy. The output of the GRU unit update gate is:

(14) $$z_{t}=\sigma \left(\omega_{z}\times \left[h_{t-1},x_{t}\right]\right)$$

(15) $$r_{t}=\sigma \left(\omega_{r}\times \left[h_{t-1},x_{t}\right]\right)$$

(16) $$h_{t}^{\prime }=\tan h\left(\omega_{t}\times \left[r\cdot h_{t-1},x_{t}\right]\right)$$

(17) $$h_{t}=\left(1-z_{t}\right)\times h_{t-1}+z_{t}\times h_{t}^{\prime }$$

In Equations (14)–(17), σ is the activation function, and tanh() is the activated hyperbolic tangent function. $z_{t}$ and $r_{t}$ are the update gate and the reset gate, respectively. $x_{t}$ is the input, and $h_{t-1}$ is the output of the previous GRU unit. $h_{t}^{\prime }$ is the information contained in $h_{t-1}$ and $x_{t}$, and $h_{t}$ is the final remaining life prediction output of the GRU unit.

**Figure 3:**
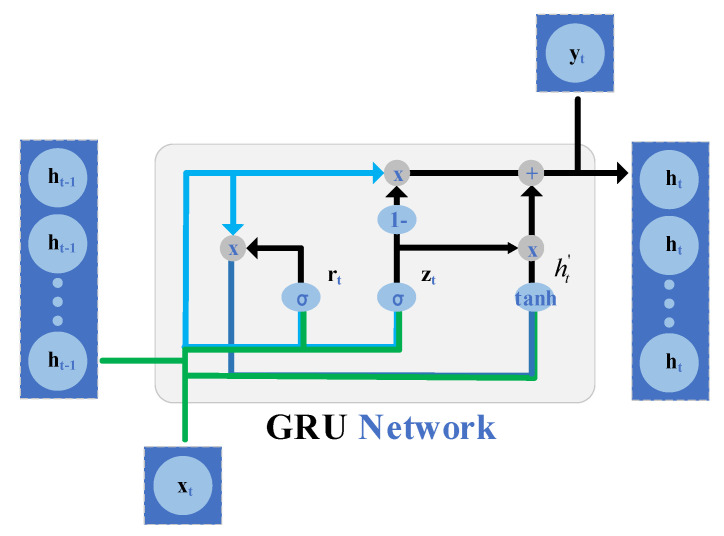
The unit internal structure of GRU.

The structure of the GRU unit is shown in Figure 3. The hidden layer state $h_{t-1}$ and the input $x_{t}$ can generate the gating signal $r_{t}$ under the action of σ. Based on this, a new state $h_{t}^{\prime }$ containing the relevant information of the input is obtained. The hidden layer state $h_{t-1}$ and the input $x_{t}$ can generate an updated gating signal $z_{t}$ under the action of σ. The gating signal is divided into two parts: $z_{t}$ and $1-z_{t}$, and the update state is selected by $h_{t}^{\prime }$ and the hidden state $h_{t-1}$ of the previous moment. On the basis of this, the hidden state $h_{t}$ of the current moment is obtained, which can be used for the output of the current moment and the input of the unit at the next moment.

**Figure 4:**
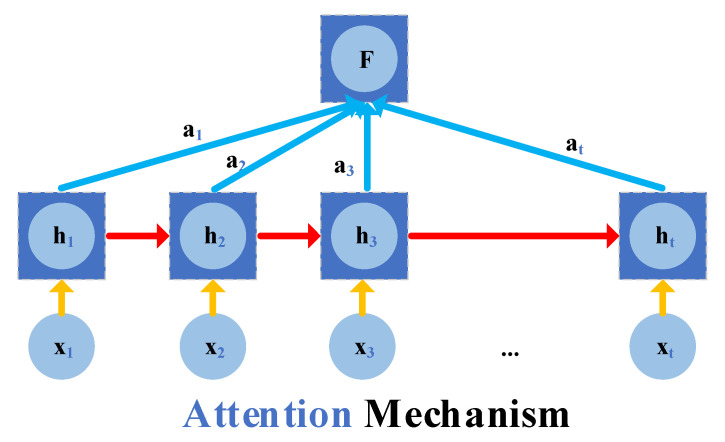
The architecture of the attention mechanism.

The attention mechanism simulates the characteristics of people paying attention to things, assigning different weights to different processing areas. Based on this, more attention will be paid to important content (distributing large weights), and weights will be reduced to the less important content. The differentiated assignment improves the quality of feature extraction and makes processing more efficient. Its working mechanism is described in Figure 4, and the concrete calculation process is formulated in Equations (18)–(20):

(18) $$F=\sum_{i=1}^{t}a_{i}\times h_{i}$$

(19) $$a_{i}=\frac{\exp\left(e_{i}\right)}{\sum_{k=1}^{t}\exp\left(e_{k}\right)}$$

(20) $$e_{i}=v_{a}^{T}\tan h\left(W_{i}h_{i}+b_{i}\right)$$

Equations (18)–(20) refer to the vector F that represents the weight sum of each hidden state in the new hidden state and the input hidden state. $e_{i}$ refers to the amount of information contained in the hidden state at the current moment. Both $v_{a}^{T}$ and $W_{i}$ are weight vectors. The network will be initialized, and the parameters in the formula will be continuously updated so that the attention state changes accordingly.

### 4.3. AR-Assisted Maintenance

In this section, the research on the human-machine interaction (HMI) in the IoT-enabled manufacturing workshop will be focused on. Meanwhile, AR is adopted to realize HMI through the feature recognition algorithm.

**Figure 5:**
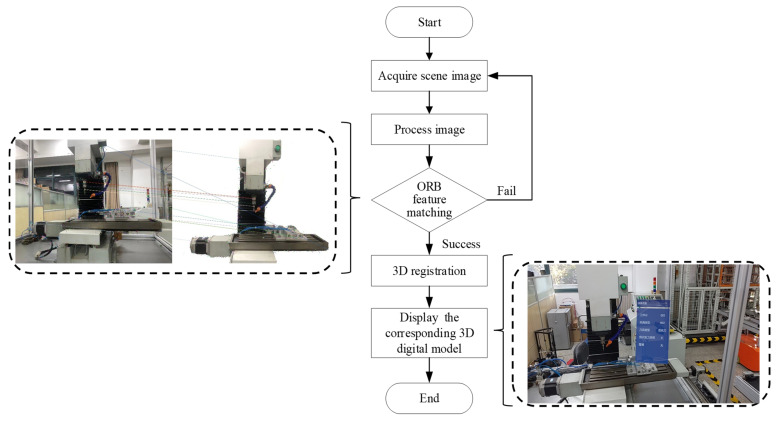
Flow chart of tracking and registration.

The IoT-enabled manufacturing workshop needs to establish a virtual three-dimensional digital model of the process in advance and provide a calibration sample of the object. On the basis of this, machines in the IoT-enabled manufacturing workshop will be associated with the digital model through registration. When the personnel with AR glasses need to identify a target object, such as machine equipment, mechanical parts, or operating tools, the tracking and identification function of the AR glasses will be triggered. The AR glasses will continuously acquire the image of the current environment at a certain frequency and process the image based on the ORB feature matching algorithm. If the matching fails, the scene image will be re-acquired. If the matching is successful, the corresponding virtual 3D digital model will be visualized. The flow chart of tracking identification is described minutely in Figure 5. Firstly, the image is acquired through AR glasses. After the image is grayed and processed, ORB is used to perform feature point matching. If the match is successful, 3D registration is performed, and accurate virtual-to-real mapping of virtual instructions is performed.

The method of intensity centroid is adopted by ORB to measure the angle change of object rotation. In the process of rotation, the center of the circle is fixed. The angle of rotation can be determined according to the change of the center of mass during the rotation process, thereby updating the coordinate system. The specific calculation formula is as follows:

(21) $$m=\sum_{x,y}x^{p}y^{q}I\left(x,y\right)$$

(22) $$m_{x}=\sum_{x,y}x^{p}y^{q}xI\left(x,y\right)$$

(23) $$m_{y}=\sum_{x,y}x^{p}y^{q}yI\left(x,y\right)$$

(24) $$C=\left(\frac{m_{x}}{m},\frac{m_{y}}{m}\right)$$

where p and q represent the boundaries of the two-dimensional image, respectively. Meanwhile, I(x,y) denotes the gray value at (x,y).

## 5. Case Study and Numerical Analysis

### 5.1. Experiment Platform and Parameters Setting

#### 5.1.1. Experiment Platform

**Figure 6:**
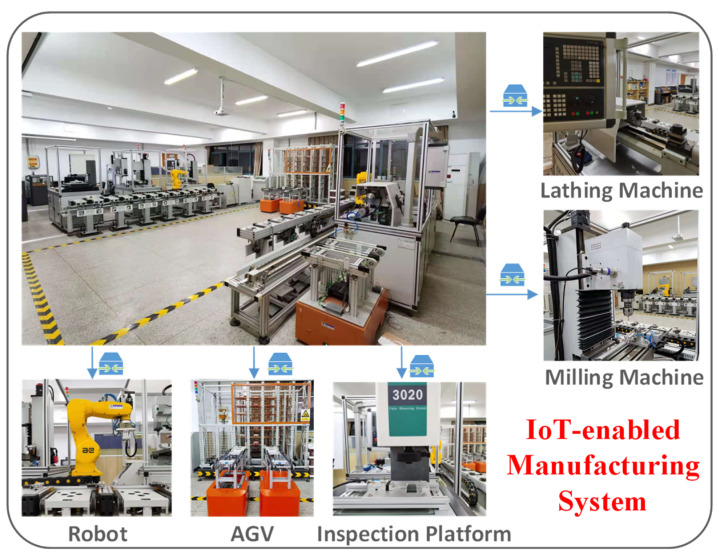
Layout of the typical machining workshop supported by the Internet of Things.

A layout of the typical machining workshop supported by the Internet of Things is described in Figure 6, which is located in Nanjing, China. The typical machining workshop consists of four machine tools: two AGVs and two robots. Based on this, the axis, flange, and boards can be processed in this workshop. Various sensors (e.g., AE sensors, vibration sensors, and force sensors) are implemented in this workshop to acquire production data. The typical machining workshop can process various personalized custom parts, such as customized shaft parts, customized flange parts, and customized plate parts.

#### 5.1.2. Parameters Setting

The adjustment results of GRU layers are shown in Table 1. It shows that if the amount of GRU layers is two, better prediction accuracy can be achieved in a short running time. If the amount of GRU layers is one, the effect is poor because the fault features can not be well analyzed, and the RUL characteristics can not be extracted. Meanwhile, if the amount of GRU layers is three, although the accuracy difference is small, the training time is too long. Additionally, if the amount of GRU layers is four, overfitting occurs, and the training time is also too long. Therefore, the optimal amount of GRU layers is set to two.

The number of network layers, the number of neurons, the dropout rate, the learning rate, and the batch size are used as the individual position of the sparrow, and the root mean square error (RMSE) between the prediction model and the actual result is used as the fitness (i.e., the food of the sparrow). Based on this, the performance of the RUL prediction model can be optimized by setting the optimal hyperparameters.

The specific calculation process of the Sparrow Search Algorithm (SSA) is described as follows:

The finder’s location update formula can be obtained as follows:

(25) $$x_{i,d}{}^{\left(t+1\right)}=\left\{ \begin{array}{l} x_{i,d}{}^{\left(t\right)}\cdot \exp(\frac{-i}{\alpha \cdot iter_{\max}}),R_{2}<ST\\ x_{i,d}{}^{\left(t\right)}+Q,R_{2}\geq ST \end{array}\right.$$

where $x_{i,d}{}^{\left(t\right)}$ represents the d-dimension location of the *i*-th sparrow in the *t*-th generation, and α is a random value in (0,1]. Meanwhile, *Q* is a random standard normal distribution number, and $R_{2}$ is a uniform random number in [0,1]. Additionally, $iter_{\max}$ is the total number of iterations, and ST is the warning threshold.

The follower’s position update formula is described as follows:

(26) $$x_{i,d}{}^{\left(t+1\right)}=\left\{ \begin{array}{l} Q\cdot \exp(\frac{xw_{d}{}^{\left(t\right)}-x_{i,d}{}^{\left(t\right)}}{i^{2}}),i>\frac{N}{2}\\ xb_{d}{}^{\left(t\right)}+\frac{1}{D}\sum_{d=1}^{D}\left[rand\{-1,1\}\cdot (\left|xb_{d}{}^{\left(t\right)}-x_{i,d}{}^{\left(t\right)}\right|)\right],i\leq \frac{N}{2} \end{array}\right.$$

where $xw_{d}{}^{\left(t\right)}$ is the position of the sparrow with the worst fitness in the current population, and $xb_{d}{}^{\left(t\right)}$ is the position of the sparrow with the worst fitness in the current population.

The alerter randomly selects A sparrows from among all individuals in each generation. They need to abandon their current location and move to a new location. The position update formula of the alerter is described as below:

(27) $$x_{i,d}{}^{\left(t+1\right)}=\left\{ \begin{array}{l} xb_{d}{}^{\left(t\right)}+Q\cdot (x_{i,d}{}^{\left(t\right)}-xb_{d}{}^{\left(t\right)}),f_{i}{}^{\left(t\right)}\neq f_{b}{}^{\left(t\right)}\\ x_{i,d}{}^{\left(t\right)}+K\cdot (\frac{x_{i,d}{}^{\left(t\right)}-xw_{d}{}^{\left(t\right)}}{\left|f_{i}{}^{\left(t\right)}-f_{w}{}^{\left(t\right)}\right|+\varepsilon }),f_{i}{}^{\left(t\right)}=f_{b}{}^{\left(t\right)} \end{array}\right.$$

where K is a uniform random number in [0,1], ε is a small enough number introduced to prevent the denominator from being 0. Meanwhile, $f_{w}$ is the worst fitness of the current population, and $f_{b}$ is the fitness of the best individual.

**Figure 7:**
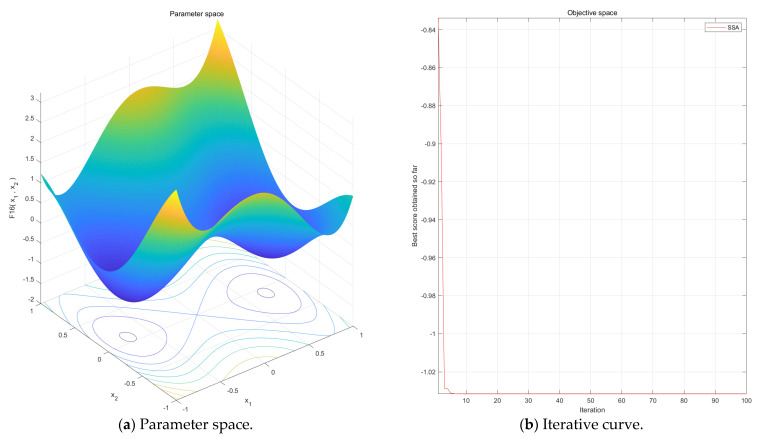
The optimal fitness update process of each generation in the optimization based on SSA.

It can be seen from Formula (27) that if the alerter is the optimal individual, it will flee to a position near itself. Otherwise, it will move towards the optimal individual. SSA is applied to optimize hyperparameters to search for high-quality hyperparameter combinations. The optimal fitness update process of each generation in the optimization is shown in Figure 7. Based on SSA, the specific parameters setting of the proposed RUL prediction model is shown in Table 2. By adjusting the setting value of dropout, over-fitting can be effectively prevented. Based on this, the probability of each neuron being discarded in dropout can be adjusted, and the generalization ability of the model can be adjusted. When the dropout set in this paper is 0.5, the generalization ability of the model is optimal, which can effectively prevent over-fitting. The specific values are shown in Table 2. All the codes are accomplished through Matlab 2019a (Cleve Barry Moler, USA)which is running on a workstation with i9-10850K CPU and NVIDIA Quadro P5000 GPU.

### 5.2. Experimental Results and Performance Analysis

#### 5.2.1. RUL Prediction Result and Performance Analysis

**Figure 8:**
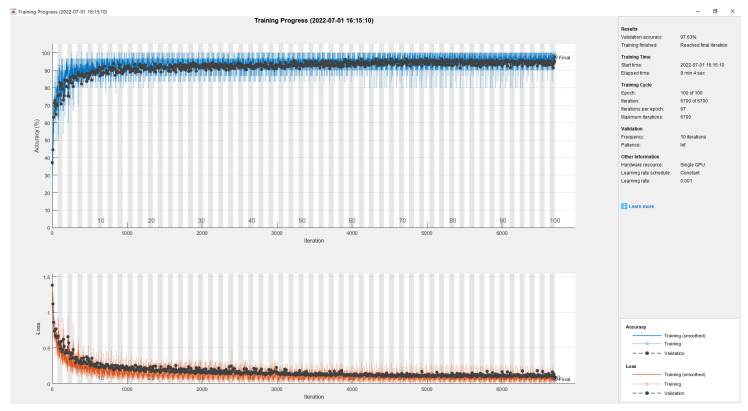
The training process of the RUL prediction model based on the proposed deep learning.

The training process of the RUL prediction model based on the proposed deep learning is shown in Figure 8. Before the first 30 epochs, the accuracy is low, and the loss is poor. After training for 100 epochs, the training process indicates that the model achieves high-accuracy prediction results. Finally, the validation accuracy reaches 97.63%.

**Figure 9:**
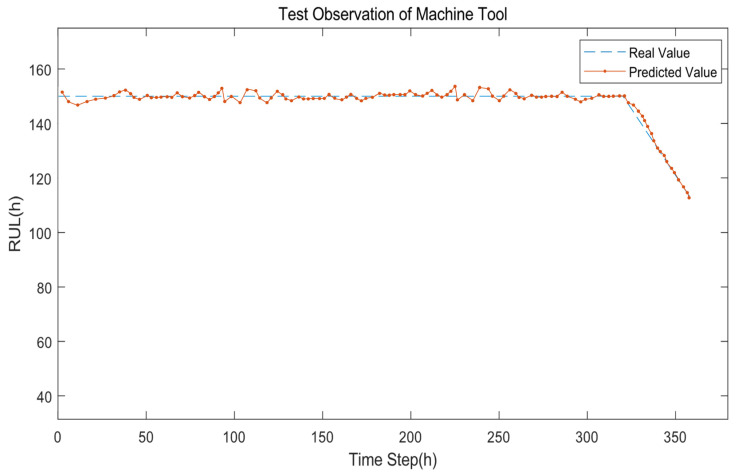
The comparison of the real RUL value and the predicted RUL value of the machine tool.

**Figure 10:**
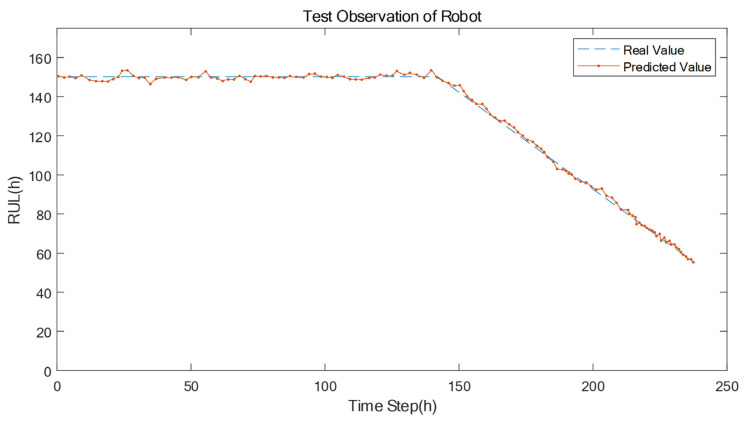
T The comparison of the real RUL value and the predicted RUL value of the robot.

**Figure 11:**
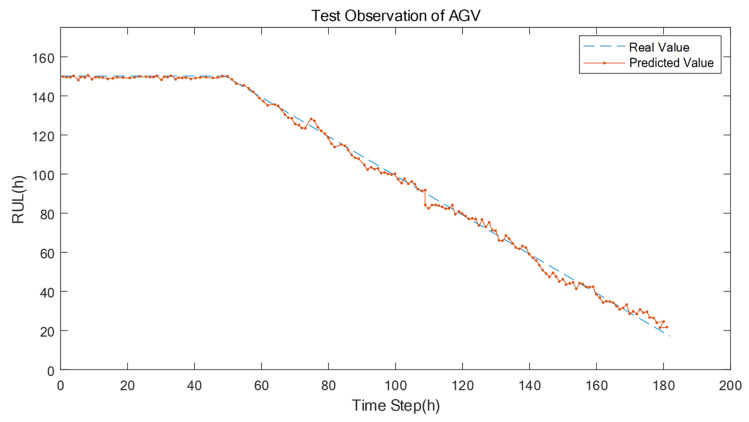
The comparison of the real RUL value and the predicted RUL value of the AGV.

The RUL prediction results based on the proposed approach are described in Figure 9, Figure 10 and Figure 11, respectively. Figure 9 demonstrates the RUL prediction result of the machine tool, where the predicted value (the orange line) slightly fluctuations around the real value (the blue line). This shows that the RUL degeneration trend of the predicted results is on the verge of the RUL degeneration trend of the actual results, which indicates that the proposed prediction model has learned the life degradation law of the machine tools. Similarly, in Figure 10 and 11, the prediction model has learned the life degradation law of the robot and AGV, respectively, so the prediction accuracy is also high.

#### 5.2.2. AR-Assisted Maintenance Result

**Figure 12:**
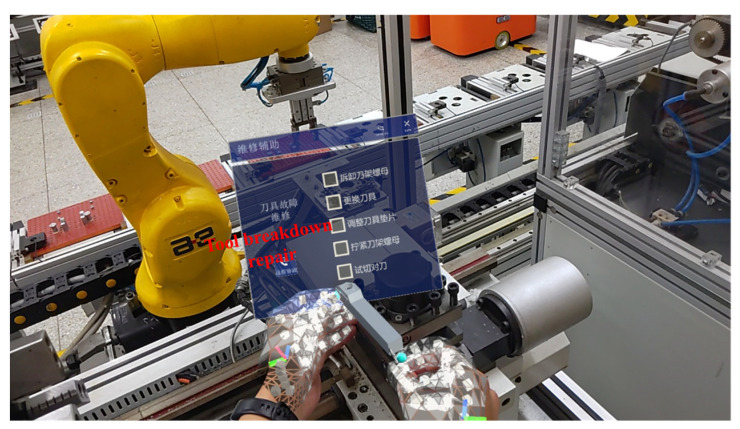
AR-assisted maintenance for the machine tool.

**Figure 13:**
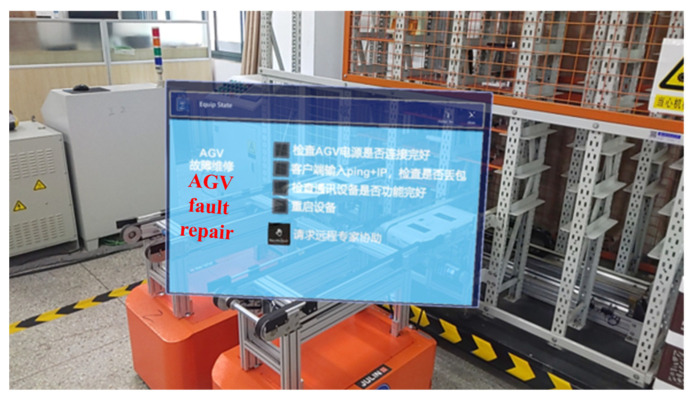
AR-assisted maintenance for the AGV.

**Figure 14:**
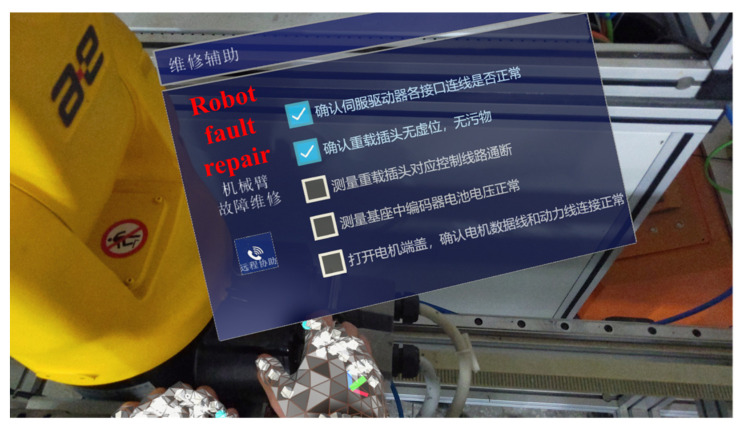
AR-assisted maintenance for the robot.

AR-assisted maintenance results of the machine tool, the AGV, and the robot are described in Figure 12, Figure 13 and Figure 14, respectively. The AR-assisted maintenance for the machine tool is described in Figure 12, and the 3D model of the machine tool appears on the parts to be repaired in the visible form. Based on this, maintenance personnel receives information on the location and condition of the parts to be repaired. The AR-assisted maintenance for the AGV is shown in Figure 13; the visible maintenance guidance is integrated into the physical world with the help of AR. Due to this, maintenance personnel can directly maintain the AGV. After completing each step, they can tick the finish button to confirm the completion of this step, which eliminates barriers to reading paper maintenance instructions. The AR-assisted maintenance for the robot is described in Figure 14, and maintenance personnel can repair the faulty joints of the robot smoothly with the help of AR-assisted visible guidance.

#### 5.2.3. AR-Assisted Remote Maintenance

**Figure 15:**
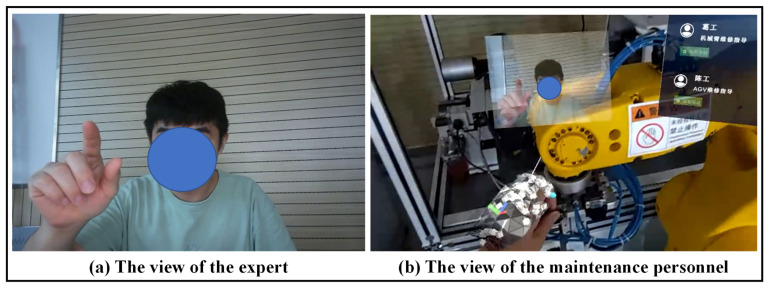
AR-assisted remote maintenance.

The remote expert system mainly occurs when the difficulty of the current task exceeds the technical ability of the maintenance personnel and cannot be successfully executed, which can be seen in Figure 15. The AR system builds a video call with a remote expert in the local area network through the WebRTC protocol. Under the guidance of the remote expert, the problem is checked step by step. After the remote expert finds the problem, they will inform the maintenance personnel in the form of annotation to assist the maintenance personnel in continuing the maintenance operation.

## 6. Discussion

### 6.1. RUL Prediction Accuracy Comparison

**Figure 16:**
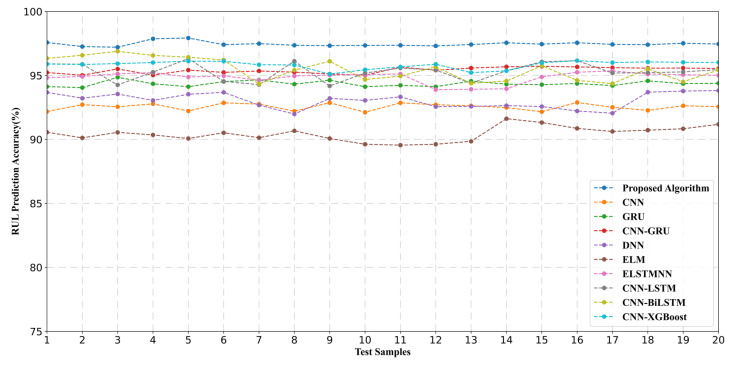
RUL prediction accuracy comparison.

For the purpose of validating the effectivity of the proposed RUL prediction algorithm, comparative experiments are implemented with CNN [39], GRU [40], CNN-GRU [41], DNN [42], ELM [43], ELSTMNN [15], CNN-SLTM [26], RBM-LSTM [18], and CNN-XGBoost [19]. This experiment contains 20 random test samples (including the remaining life datasets of the machine tool, the robot, and AGV), and each sample set is subjected to 100 experiments to calculate the prediction accuracy. RUL prediction accuracy comparison results are shown in Figure 16, which indicates that the proposed algorithm (the blue line) is obviously superior to the other comparative algorithms. Meanwhile, the result of CNN-GRU (the red line) is slightly worse than the proposed algorithm (the blue line) due to the lacking of the attention mechanism. The result of CNN-GRU (the red line) is superior to the result of CNN (the yellow line), which indicates that CNN has a strong feature extraction ability. Compared with ELM, the result indicates that deep learning algorithms have better mining and fitting capabilities of data associations than the general machine learning. The method mentioned in the literature [26] focuses on the maintenance personnel scheduling algorithm based on deep reinforcement learning. However, this paper focuses on the improvement of the model structure of the RUL algorithm, which aims to provide a more accurate basis for the subsequent maintenance plan. In addition, the equipment objects in the experiment in this paper are different from the objects predicted by RUL in the method mentioned above. Therefore, in the research scenario of this paper, the accuracy of the proposed RUL prediction model is slightly higher than the method mentioned above, which can be seen in Figure 16. The complete average accuracy comparison results are described minutely in Table 3, which verifies that the proposed algorithm has optimal performance for RUL prediction.

### 6.2. Advantages of AR-Assisted PHM

With the aim of verifying the advantages of AR-assisted PHM, three real maintenance cases with various difficulty levels are implemented in the typical machining workshop supported by the Internet of Things, which is shown in Table 4. Case No.1 represents the AGV battery replacement, and the difficulty level of the maintenance task is easy. Case No.2 represents the tool change of the machine tool, and the difficulty level of the maintenance task is medium difficulty. Moreover, case No.3 represents the drive board replacement of the robot and demonstration, which requires skillful and experienced personnel to repair it. Therefore, the difficulty level of the maintenance task is complex.

**Figure 17:**
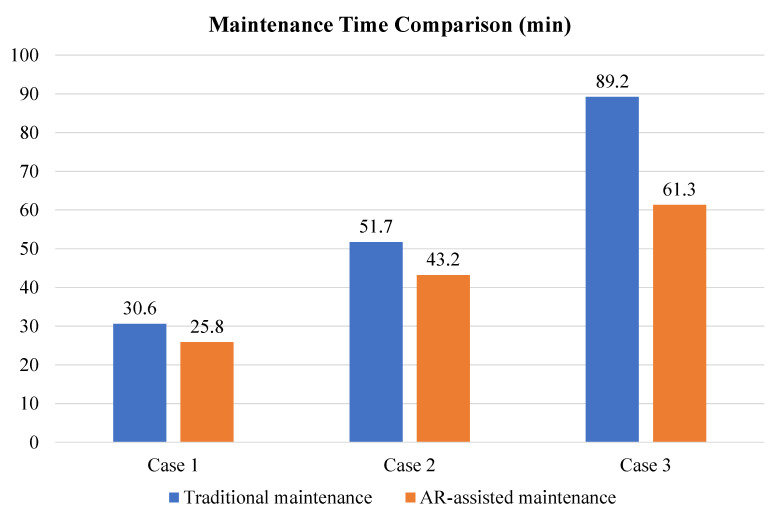
Advantages of AR-assisted PHM.

The advantages of AR-assisted PHM are described in Figure 17 minutely. It is obvious that AR-assisted maintenance (the orange histogram) is superior to traditional maintenance (the blue histogram) in terms of maintenance time. In Case No.1, the difference in the maintenance time is not very big because the maintenance task is not too difficult. However, the difference in the maintenance time is obviously big in Case No.3 because the maintenance task is complex. That is to say, AR offers great help in improving maintenance efficiency due to visible guidance, especially in complex maintenance tasks.

## 7. Conclusions

In this research work, an augmented reality-assisted prognostics and health management system based on deep learning for IoT-enabled manufacturing is proposed. For the purpose of achieving high-precision RUL prediction results as the backbone of reliable maintenance, PSO-CNN is implemented to accomplish high-efficiency feature extraction from the vast production data, and PSO is used to find the optimal value by initializing a group of particles and continuously updating the speed and position. Based on this, GRU-attention is constructed to mine the associations between these features and fit the RUL result. Due to the attention mechanism, more user attention will be applied to vital content. Based on the RUL prediction result, AR-assisted maintenance is applied for the visible guidance of the maintenance personnel. Additionally, the AR remote expert can support guidance in the form of annotation to assist the maintenance personnel in continuing the maintenance operation. The experiment results demonstrate that both the accuracy and the efficiency are superior to other comparative methods.

In the future, more machines will be involved in the IoT-enabled manufacturing workshop. Thus, the cloud-edge orchestration mechanism should be integrated into the proposed approach in this paper. The RUL prediction model can be deployed on each industrial computer of the machine tools in the edge, and the maintenance personnel scheduling algorithm can be deployed in the cloud. Based on this, suitable maintenance personnel can be more precisely assigned to the corresponding task with the help of AR, and maintenance efficiency can be further improved. Thus, future research can focus on the following questions. (1) How to employ the RUL prediction model through the cloud-edge orchestration mechanism to achieve both high accuracy and high computation efficiency? (2) How to arrange a suitable maintenance scheme based on the predicted RUL?

## Figures and Tables

**Table 1:** The optimal parameters setting of the GRU-attention model.

Layers of GRU	Neurons	Epochs	Batch Size	RMSE/%	Running Time/s
1	64	100	128	1.96	527
2	64	100	128	1.03	544
3	64	100	128	1.37	689
4	64	100	128	2.59	856

**Table 2:** Parameters setting of the proposed RUL prediction model.

Parameters	Description	Value
LR	Learning rate.	0.001
Kernel_size Padding	Kernel size of the CNN layer. The padding method of the CNN layer.	3*3 Same
Pooling	The pooling method of the CNN layer.	Max pooling
Dropout	Dropout of the model.	0.5
PSO_fitness	The fitness function of PSO.	RMSE_CNN
Epoch	The total training epochs of the model.	100
Maximum_iterations	Maximum iterations of the model.	6700
Optimization	The optimization method of the model.	Adam
Batch_size	The batch size of the model.	128
Loss_function	The loss function of GRU-attention.	RMSE
Activate_function	Activate function.	ReLU

**Table 3:** Average accuracy of the comparative algorithms.

Algorithm	Average Accuracy
CNN	92.56%
GRU	94.39%
CNN-GRU	95.61%
DNN	94.68%
ELM	90.37%
ELSTMNN	95.12%
CNN-LSTM	95.27%
CNN-BiLSTM	95.83%
CNN-XGBoost	96.02%
Proposed algorithm	**98.16%**

**Table 4:** Maintenance cases.

Case	Maintenance Task	Difficulty Level
No.1	AGV battery replacement.	Easy
No.2	Tool change of the machine tool.	Medium difficulty
No.3	Drive board replacement of the robot and demonstration.	Complex

## Data Availability

Raw data were generated at Navicat 15 for MySQL. Derived data supporting the findings of this study are available from the corresponding author Liu on request.
